# From Principles to Practice: One Local Health Department's Journey Toward Health Equity

**DOI:** 10.1089/heq.2016.0004

**Published:** 2017-01-01

**Authors:** Umair A. Shah, Jennifer M. Hadayia, Linda E. Forys

**Affiliations:** ^1^ Harris County Public Health, Houston, Texas.

**Keywords:** health disparities, health equity, public health

## Abstract

Few dispute that social determinants such as economics, education, and the environment are the true drivers of health. In fact, the recent “Public Health 3.0” publication is a national call to action for public health to focus upstream. Where there is less clarity is how to redesign public health practice to address social determinants. As an example of how local health departments can heed this national call, Harris County Public Health describes its movement from health equity principles to practice, which included reframing an understanding of health inequities and applying a multitiered infrastructure of policy and procedures for “retrofitting” practice.

## Introduction

Earlier this year, some of our nation's highest ranking health officials issued a national call to action to “boldly expand the scope and reach of public health.”^1^ They appealed to all state and local public health agencies to make an *upgrade* to Public Health “3.0,” a modern understanding of public health that deliberately shifts attention upstream to the true drivers of health, which, the authors note, are not healthcare services, but the social determinants of health such as economics, education, and the environment.^1^

For those in local public health who have been taking a deeper dive into the social determinants for some time, this was a welcome wake-up call. Six years have passed since *Healthy People* declared social determinants as one of our nation's leading health indicators, and it has been more than a decade since the World Health Organization (WHO) established the Commission on Social Determinants of Health and shortly after issued its landmark report on closing the gap in social factors.^4–6^ In 2016, the national associations for state and local public health focused their attention on closing the gap in social determinants and achieving health equity.^4–6^ Public Health 3.0 signaled a long overdue critical mass in public health leadership, one that has officially declared health equity mission critical.

Where there is less clarity and consensus in the field is how to redesign state and local public health to address social determinants in day-to-day practice. Harris County Public Health offers an example as we have been deliberately moving from health equity principles to practice since 2014, including reframing our understanding of how health inequities occur (our Health Equity Framework) and developing policy and procedures for “retrofitting” public health practice upstream (our Health Equity Infrastructure). We are now focused on operationalizing a health equity lens into all of our public health services.

## In Harris County

Harris County, Texas, is the third most populous county in the United States with 4.1 million residents.^5^ It is home to the fourth largest city in the country (Houston), the world's largest nonprofit medical center (Texas Medical Center), one of the world's largest shipping ports (Port of Houston), and the largest concentration of chemical manufacturing and petroleum refining facilities in the nation. All of this creates a complex mixture of health assets and risks.

In terms of the social determinants of health, Harris County faces some challenges. It has a higher rate of both poverty (17%) and achievement gap (20%) than the United States as a whole.^7^ There are also strong correlations between poor health and, independently, race, lack of health insurance, lower income, and less education.^8^ The relationship between socioeconomic conditions and health also holds at the subcounty level with hardship and poor health occurring in the same neighborhoods.^9^

As the county health department, we are concerned with the health of the entire population of Harris County. When faced with the mentioned data, it was impossible to ignore that a status quo approach to health, one that focuses primarily on preventing disease and injury in Harris County, would not move the needle on disease and injury, at least not for all people in Harris County. Instead, to ensure population health improvement, we needed to focus further upstream. Thus, we began a deliberate transformation toward the practice of health equity both in our organization and in our community. The first step was to include health equity as a priority in our Agency Strategic Plan. We then launched health equity activities in four domains: workforce development, policy and procedure, performance management, and public narrative. We adopted the following definition of health equity to guide our work: “health equity is a state in which every person has the opportunity to attain his or her full health potential and no one is disadvantaged from achieving this potential because of socioeconomic or environmental conditions.”^10^

## A Health Equity Framework

**FIG. 1.**
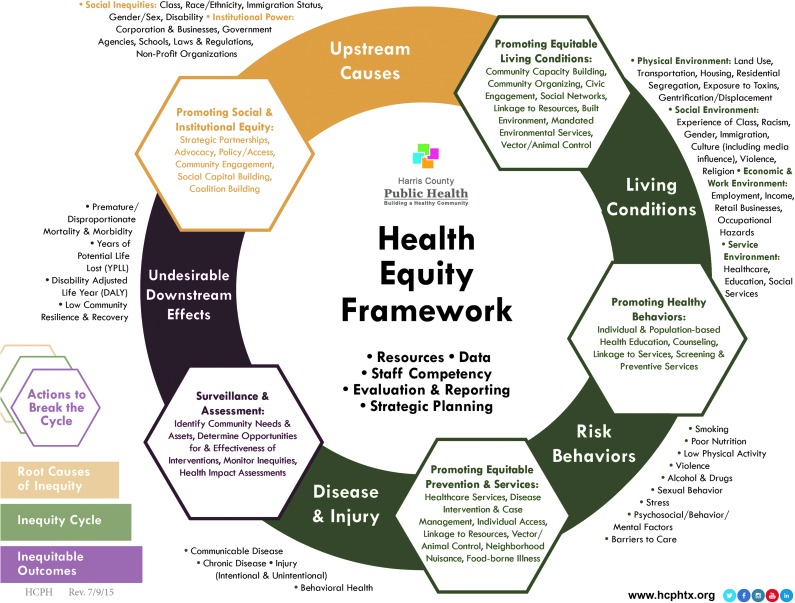
Health Equity Framework for Harris County. The Harris County Public Health's Health Equity Framework shows our understanding of the trajectory of health inequities beginning with Upstream Causes and leading to Undesirable Downstream Effects. It also concurrently shows the Public Health Actions that help break the health inequity cycle at each step in the trajectory. *Yellow*, root causes of inequity; *green*, inequity cycle; *red*, inequitable outcomes. *Cycle components:* steps in trajectory of health inequities, beginning with upstream causes of social conditions and institutional practices (*yellow*); followed by living conditions, risk behaviors, and disease and injury (*green*); and ending with undesirable downstream effects (*red*). These are presented as a cycle to represent how downstream morbidities also contribute to upstream causes. On the outside of the cycle, next to each color-coded component, are lists of the specific social, structural, and other determinants that relate to each component. *Hexagons:* identified public health actions that help break the health inequity cycle at each component. Inside each hexagon is a list of specific tasks related to the particular component of the cycle. *Center of cycle:* a list of cross-cutting assets that support health equity. Adapted from the Bay Area Regional Health Inequities Initiative (BARHII).^11^

Shifting long-standing approaches in public health practice to the social determinants of health requires a commonly held understanding of how upstream factors actually lead to downstream inequities. We developed a health equity framework specific to our health department (Fig. 1), which shows how the upstream causes of social conditions and institutional practices can create inequitable living and working conditions, which, in turn, lead to risk behaviors, disease and injury, and, ultimately, reduced length and quality of life (the undesirable downstream health effects). We based our framework on the pioneering health equity framework developed by the Bay Area Regional Health Inequities Initiative (BARHII).^11^ Uniquely, our framework also recognizes that disproportionate downstream morbidities can essentially “restart” root causes of health inequities by further disconnecting populations from social and economic resources.^12^ Different from the BARHII framework, we posit health inequities to be a causal loop instead of a causal chain. In addition, our framework integrates the specific local public health actions that can “Break the Cycle” of health inequities in Harris County. These actions encompass all areas of our health department and are where our practice redesigns begin.

## A Health Equity Infrastructure

With our health equity framework in place, two key questions emerged: will staff and programs have the guidance they need to transform practice in the direction of social determinants and how will we know when health equity goals have been met? We addressed these questions by developing a three-tiered health equity policy and procedure portfolio that now serves as our organizational infrastructure for health equity practice (Table 1). At the top of the infrastructure are high-level expectations (policy) that define success for our agency: that a health equity lens will be applied to programming, assessment, communications, data, workforce, partnerships, and resources. We have assigned quantitative measures to each of these categories, which, together, form a health equity “dashboard” for our agency. The midlevel provides hands-on guidance to staff and programs for meeting these expectations in the form of health equity procedures, or step-by-step protocols for identifying and implementing health equity “retrofits” in each of the areas already listed. Last on the infrastructure (and closest to day-to-day practice) is a work plan template for outlining how each health department unit can plan and move its work further upstream.

**Table 1.** **A Structure for Health Equity Practice in Local Public Health**

Level	Document	Content
Macro	Health equity policy	Agency-wide expectations for where a health equity lens should be applied such as programming, assessment, communications, data, workforce, partnerships, and resources.
Mezzo	Health equity procedures	Step-by-step instructions for how to achieve macrolevel expectations using the cross-cutting strategies of program design, community engagement, strategical partnerships, data management (collection, analysis, reporting, monitoring, and evaluation), and communications.
Micro	Health equity work plan	A checklist for identifying and documenting implementation of health equity improvements to programs and practice.

## From Health Equity Principles to Practice

We adopted our health equity framework in 2014 and launched our health equity infrastructure in 2016. In less than 6 months, these steps have already produced tangible changes in our approach to public health practice in each area of the health inequity cycle (Table 2). A particularly emergent example is how we applied health equity in our response to the public health emergency of Zika. As cases of Zika and its associated neonatal anomalies spread through South America in early 2016, we designed a response plan for Harris County through a health equity lens.

**Table 2.** **Examples of Changes to Practices at Harris County Public Health That Break the Cycle of Health Inequities**

Precipitating factor	Action to break the cycle of health inequity	Examples of health equity practice at Harris County Public Health
Upstream causes	Promote social and institutional equity	Formal evaluations of proposed state legislation (bills) include a review of their health equity impact.
		Health Impact Assessments (HIAs) of proposed institutional decisions are conducted throughout the county. The HIAs process includes a significant health equity component.
Living conditions	Promote equitable living conditions	Place-based projects are launched that focus on improving the built environment in disinvested neighborhoods. One example is the BUILD Health Partnership working to change the food environment in the city of Pasadena, Texas.
		Partners are convened in a Collective Impact model to work on policy-level improvements to living conditions countywide. One example is Healthy Living Matters working on issues such as community safety and land use.
Risk behaviors	Promote healthy behaviors	Behavior change interventions are provided in communities with specific upstream vulnerabilities such as lack of health insurance.
Disease and injury	Promote equitable prevention services	The healthcare sector is being engaged to incorporate screening for and referral to services that address the social determinants of their patients.
Undesirable downstream effects	Surveillance and assessment	Certain disease surveillance data are analyzed according to a health equity methodology that identifies disease trends in the county by income.
		The internal agency performance management dashboard includes metrics of upstream causes for Harris County such as poverty and disability.

Harris County Public Health is the county health department in Harris County, Texas.

From our health equity framework, we knew that the unhealthful environments that exacerbate Zika virus spread are inequitably distributed in Harris County and that such living conditions are caused by historical social and economic inequities. With this new understanding, we used subcounty social and economic indicators such as social vulnerability, poverty, and illegal dumping as criteria for mosquito abatement strategies, including where to place mosquito traps. From the application of our health equity policy and procedures, we developed a Zika communications plan that prioritizes populations experiencing historical inequities such as the homeless and those living in economically disinvested neighborhoods. Repellent and other prevention modalities are distributed to these vulnerable groups.

A traditional response to an infectious disease outbreak may not consider the causal loop of health inequities such as historical neighborhood-level social and economic vulnerabilities. With our health equity elements in place, however, we were able to (re)design our traditional Zika response into one that is both informed by and addresses the social determinants of health in Harris County.

## Conclusion

Few dispute that long-term population health improvement requires change in the social determinants of health, particularly the upstream factors of economics, education, and environment. National public health associations have held conferences and issued official challenges to help make this bold shift in public health practice happen. For Public Health 3.0 to become a reality, state and local public health agencies will need to move synergistically, effectively, and swiftly from (health equity) theory to practice.

Harris County Public Health had the great benefit of learning from early pioneers in the field, and to these pioneering approaches we have added practical tools to help our staff and programs “retrofit” their local public health practice even further upstream. Our health equity framework and policy and procedure infrastructure are guiding staff day-to-day in making equity-focused improvements to public health programming, assessment, communications, data, workforce, partnerships, and resources. Organizationally, this work is transforming public health practice in Harris County and forging new pathways of innovation and community engagement along the way. We believe our approach is replicable to other local public health agencies and, if applied, can help generate the bold expansion of scope and reach of public health as a field that has been long overdue. We hope others join us on this collective journey to Public Health 3.0.

## Abbreviation Used

BARHII: Bay Area Regional Health Inequities Initiative
